# Emergence of dual drug-resistant strongylids in goats: first phenotypic and genotypic evidence from Ratchaburi Province, central Thailand

**DOI:** 10.1186/s12917-025-04700-4

**Published:** 2025-04-04

**Authors:** Abigail Hui En Chan, Chanisara Kaenkaew, Wallop Pakdee, Sivapong Sungpradit, Urusa Thaenkham

**Affiliations:** 1 Department of Helminthology, Faculty of Tropical Medicine, Mahidol University, Bangkok, Thailand; 2 Department of Pre-Clinic and Applied Animal Science, Faculty of Veterinary Science, Mahidol University, Nakhon Pathom, Thailand

**Keywords:** Caprine strongylids, Anthelmintic resistance, *In vitro* test, Genotyping

## Abstract

**Background:**

This study provides crucial insights into the prevalence and drug resistance patterns of strongylid gastrointestinal nematodes (GINs) in goats in Thailand, highlighting resistance to albendazole and levamisole. Strongylids, particularly *Haemonchus* sp. and *Trichostrongylus* sp., pose a significant threat to goat health. With the global rise of anthelmintic resistance, the detection of multidrug resistance in Thailand’s goat population is concerning, given the frequent import and export of goats. This resistance challenges effective parasite control strategies. This study aimed to identify strongylid species using both morphological and genetic methods and to assess resistance to albendazole and levamisole through phenotypic and molecular approaches.

**Results:**

Fecal samples from 30 goat farms in Ratchaburi Province revealed a high prevalence of strongylid infection (87%), with *Haemonchus* sp. and *Trichostrongylus* sp. detected on 100% and 96% of farms, respectively. Phenotypic assays demonstrated significant drug resistance, with 90% and 71% of farms harboring strongylid populations resistant to albendazole and levamisole, respectively. Genotypic analysis of pooled infective larvae showed that 100% of farms had albendazole-resistant strongylid populations, with 31% homozygous and 69% heterozygous resistance, and *Trichostrongylus* sp. showing 48% homozygous and 52% heterozygous resistance. For levamisole resistance, 92% of farms contained resistant strongylid populations, with *Haemonchus* sp. exhibiting 11% homozygous and 81% heterozygous resistance.

**Conclusions:**

This study provides the first comprehensive evaluation of phenotypic and genotypic resistance in strongylid nematodes in Ratchaburi Province, addressing a key geographical gap in Thailand’s resistance data. The findings highlight the urgent need to reassess GIN management practices and develop sustainable strategies to mitigate resistance. Furthermore, these results have significant implications for transboundary livestock health, emphasizing the necessity of collaborative efforts to combat the growing challenge to anthelmintic drugs.

**Supplementary Information:**

The online version contains supplementary material available at 10.1186/s12917-025-04700-4.

## Background

Gastrointestinal nematode (GIN) infections pose a significant threat to ruminants, contributing to an estimated global economic loss of USD 7 billion annually [1]. Small ruminants, particularly goats, are highly susceptible to GIN infections, severely impacting productivity and overall health. Infected goats experience weight loss, decreased milk and meat yield, and general health deterioration [2, 3]. Additionally, the cost of anthelmintic treatments exacerbates economic losses, further reducing the profitability of goat farming worldwide [4].

Strongylid nematodes, including genera such as *Haemonchus*, *Trichostrongylus*, *Cooperia*,* Chabertia*, and *Oesophagostomum*, are the most common GINs infecting goats [5]. Among these, *Haemonchus contortus* and *Trichostrongylus* species pose the greatest threat to goat health [6], with *H. contortus* causing severe anemia and mortality. Sporadic cases of human infections with *H. contortus* and *Trichostrongylus* sp. have been reported in various countries such as Thailand, Lao People’s Democratic Republic, South Korea, Iran, and Australia [7–10], demonstrating their zoonotic potential, particularly in regions where humans and goats share close contact.

The emergence of anthelmintic resistance poses a significant challenge for farmers and veterinarians, impacting goat health and productivity. As resistance undermines treatment efficacy, urgent management strategies are required to sustain goat farming. Poor management practices and improper dosing further exacerbate GIN infections [11]. Resistance of strongylid nematodes to anthelmintics, including multiple-drug resistance across the three main anthelmintic classes — benzimidazoles, imidazothiazoles, and macrocyclic lactones, have been reported from Europe, Australia, and Asia [12, 13]. Detection of anthelmintic resistance traditionally relies on phenotypic methods, including the egg hatch test (EHT) and larval development test (LDT), while the evaluation of drug efficacy relies on the fecal egg count reduction test (FECRT). However, molecular approaches increasingly complement phenotypic methods, allowing for precise detection of resistance-associated alleles. For instance, benzimidazole (albendazole) resistance is identified through single nucleotide polymorphisms (SNPs) in the beta-tubulin (β-tubulin) isotype 1 gene, with quantitative PCR (qPCR) assays targeting key mutations—F200Y, F167Y, and E198A—especially in *H. contortus* [14–17]. Similarly, a qPCR assay has been developed to detect imidazothiazole (levamisole) resistance by identifying a 63 bp deletion in the *Hco-acr-8* gene of *H. contortus* isolates [18]. These molecular tools enhance genotypic diagnosis, offering valuable approaches for detecting drug-resistant alleles and improving resistance management strategies.

GIN infections in goats have been reported across several provinces in Thailand, including Nakhon Pathom, Kanchanaburi, Khon Kaen, and Satun, with studies documenting a high prevalence of strongylid infections [18–21]. Molecular analyses have confirmed the presence of *Haemonchus* sp., *Oesophagostomum* sp., and *Trichostrongylus* sp., along with benzimidazole-resistant genes in *H. contortus* [22]. Additionally, resistance to all three major anthelmintic drug classes has also been identified in Sing Buri Province [23]. These findings highlight the widespread prevalence of strongylid GINs and the escalating challenge of anthelmintic resistance in Thailand’s goat population. However, further research is needed to assess GIN infection dynamics and drug resistance in other regions, particularly in provinces engaged in importing and exporting goat kids, where disease transmission may have broader transboundary implications [24].

This study focuses on Ratchaburi Province in central Thailand, where phenotypic methods have recently reported anthelmintic resistance [25]. Our objectives are to identify the GIN species infecting goats in Ratchaburi and detect drug-resistant genes for albendazole and levamisole using phenotypic and molecular techniques. This finding will provide valuable insights for farmers and veterinarians while contributing to global efforts to manage GIN infection and combat anthelmintic resistance.

## Methods

### Ethics statement

The study on goat farms was approved by the Ethical Committee of the Faculty of Veterinary Science, Mahidol University (Approved Codes assigned by FVS-MU-IACUC: COA. No. MUVS-2023-08-54 and COA. No. MUVS-2024-03-24).

### Goat farms and sample collection

**Fig. 1:**
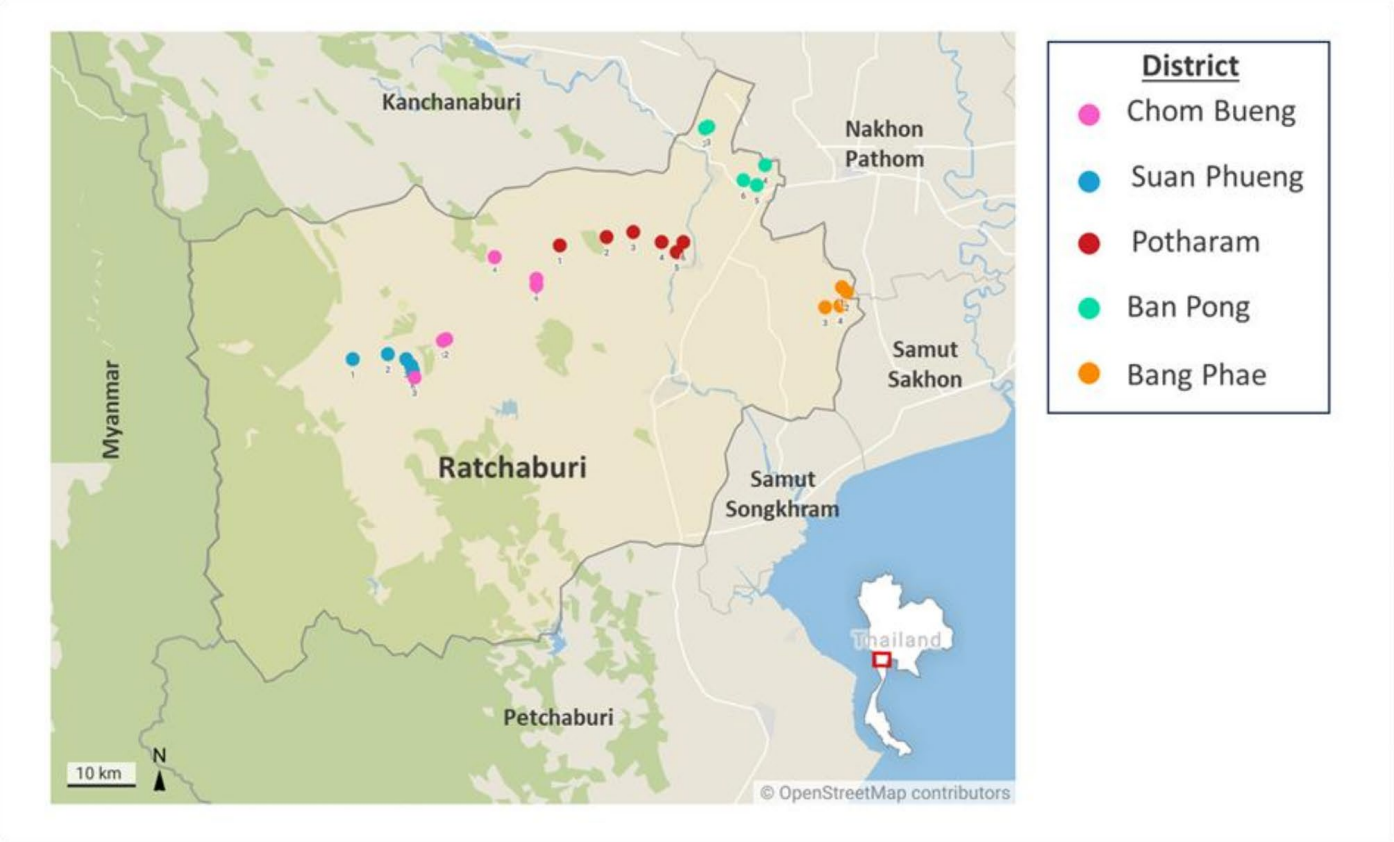
Locations of goat farms where samples were collected. The colored circles indicate the 30 farms distributed across five districts, with six per district

Fecal samples were collected from 173 goats across 30 farms in five districts in Ratchaburi Province between November 2023 (dry season) and June 2024 (wet season) (Fig. 1). The districts surveyed included Chom Bueng, Suan Phueng, Potharam, Ban Pong, and Bang Phae. The selected farms comprised both dairy and meat goat operations, with most adopting an open-system husbandry approach, where goats graze freely during the day and are housed indoors at night. The history of anthelmintic drug usage on each farm was also recorded.

Fecal samples were collected from each goat’s rectum using clean gloves and placed in 50 ml Falcon tubes. The samples were then transported to the Department of Helminthology, Faculty of Tropical Medicine, Mahidol University, Bangkok, for further analysis.

### Gastrointestinal helminth identification and quantification

#### Microscopic examination

GIN identification and strongylid egg quantification in the goat fecal samples were conduced using simple flotation and the McMaster techniques [26, 27]. Approximately 4 g of feces were homogenized with water and filtered through a sieve into a 500 ml beaker. The homogenate was allowed to sediment, and the supernatant was decanted. Saturated sodium chloride (NaCl, 56 ml) was added, and the homogenate was transferred into 15 ml Falcon tubes. Additional NaCl was then added to each tube to form a reverse meniscus at the top. A coverslip was carefully placed over the tube opening and left undisturbed for 15 min. For nematode egg identification, the coverslip was removed and placed on a microscope slide for examination under a light microscope.

For quantification, a homogenized sample from one Falcon tube was withdrawn using a glass pipette and transferred to McMaster counting chambers. The slides were left to settle for 5 min before being examined under a light microscope. Strongylid eggs were counted using a 10x objective lens, and the eggs per gram (EPG) were calculated by multiplying the total number of strongylid eggs by 50.

#### *Molecular identification*

Following microscopic examination, the eggs were washed, pooled by the farm, and preserved in 70% ethanol at − 20 °C until molecular identification of strongylid eggs. Genomic DNA was then extracted from the pooled strongylid eggs using the Genomic DNA mini kit (Geneaid Biotech Ltd., Taipei, Taiwan) according to the manufacturer’s instructions. Before DNA extraction, the eggs were homogenized with silica beads in lysis buffer using a TissueLyser LT (Qiagen, Hilden, Germany) to facilitate cell disruption.

Nested PCR was conducted using the nuclear internal transcribed spacer 2 (ITS2) region and the mitochondrial 16S  ribosomal RNA (rRNA) gene to molecularly identify strongylid eggs. Genus-specific primers targeted *Haemonchus*, *Trichostrongylus*, and *Oesophagostomum*. The ITS2 primers followed Income et al. (2021), while the first-round 16 S rRNA primers were based on Chan et al. (2020). Genus-specific primers for nested-PCR were newly designed in this study [20, 28]. Briefly, reference 16S rRNA gene sequences of *Haemonchus* sp., *Trichostrongylus* sp., and *Oesophagostomum* sp. were obtained from the NCBI database and aligned using ClustalX 2.1 [29]. Genus-specific primers were then designed within the PCR amplicon sequence from the first round of PCR. The properties (GC content, amplicon size, melting temperature, hairpin formation) and specificity of the newly designed primers were evaluated using OligoCalc version 3.27 and FastPCR [30, 31]. Table 1 summarizes the 16S genus-specific primers used in this study, along with their respective annealing temperature and amplicon sizes.

PCR amplification was carried out using a T100™ thermocycler (Bio-Rad, California, USA) in a final reaction volume of 20 µl. The mastermix consisted 10 µl of 2X i-Taq™ mastermix (iNtRON Biotechnology, Gyeonggi, South Korea), along with 2 µM of each ITS2 primer or 10 µM of each 16S primer for the first round of PCR. For the second PCR round, amplicons from the first round were diluted tenfold in nuclease-free water and used as the DNA templates, following the same mastermix preparation conditions for each genus-specific primer. The PCR thermocycling conditions were as follows: initial denaturation at 94 °C for 2 min; 35 cycles of 94 °C for 30 s, the respective annealing temperature for 30 s, 72 °C for 30 s, followed by a final extension at 72 °C for 10 min. Amplicons were examined via 1.5% agarose gel electrophoresis stained with SYBR™ safe (Invitrogen, Massachusetts, USA). Representative amplicons were then sent for Barcode Tag sequencing by a commercial provider (Celemics, Seoul, South Korea) for species-level identification.

#### *Phylogenetic analysis*

Electropherograms of the ITS2 and 16S rRNA sequences were analyzed using Bioedit 7.0 [32]. Multiple sequence alignments with reference sequences from the NCBI database were performed using ClustalX 2.1 for each genetic marker [29]. The reference sequences used are provided in Additional File 1. Phylogenetic analyses were conducted using the neighbor-joining (NJ) and maximum-likelihood (ML) methods in MEGA X [33]. To assess tree topology support, 1,000 bootstrap replicates were performed, and the best-fit nucleotide substitution model was selected for the ML analysis. *Trichuris discolor*,* Strongyloides ratti*, and *Strongyloides stercoralis* were used as outgroups to root the phylogenetic trees. The resulting trees were visualized and annotated using FigTree 1.3.1 [34].

**Table 1:** Genus-specific 16S rRNA gene primers used for molecular identification, along with their corresponding amplicon sizes and annealing temperatures

Genetic marker and genus	Primer name and sequence (5’– 3’)	Annealing temperature	Amplicon size
16S *Haemonchus*	16S Hae-F: CATATTTRATCCAGAWG	45 °C	116 bp
	16S Hae-R: CGTTAAATTAAATAAWAG		
16S *Trichostrongylus*	16S Tricho-F: CTAGGGTAGAATATTATT	41 °C	173 bp
	16S Tricho-R: AAAGAAGAACAGTCTTAAT		
16S *Oesophagostomum*	16S Oeso-F: CTTCGGAAATTCTTTTTTGG	51 °C	205 bp
	16S Oeso-R: CTTCTCCCTCTTTAACAAAC		

### Anthelmintic resistance detection

#### Phenotypic resistance detection of albendazole via egg hatch test

The egg hatch assay for detecting albendazole resistance in strongylids was performed following the protocol of Coles et al. (1992, 2006) and von Samson-Himmelstjerna et al. (2009) [35–37]. Goat fecal samples were collected and maintained under anaerobic conditions until the experiment. Briefly, the fecal samples were placed in 50 ml screw-top Falcon tubes containing 8-mm diameter glass beads and tap water. The tubes were vigorously shaken to disperse the fecal material before being transported to the laboratory. The experiment was conducted within seven days of sample collection. Each sample (5 g of feces in 42 ml of water) was homogenized, sieved, and allowed to sediment for at least 30 min. After decanting the supernatant, saturated NaCl was added to the homogenate, which was then transferred to 15 ml Falcon tubes. Additional NaCl was added to create a reverse meniscus at the top, and a coverslip was placed over the opening. The tubes were left undisturbed for 15 min before further processing.

To isolate the eggs, coverslips were rinsed with distilled water, and approximately 100 eggs in 2 ml of water were transferred to each well of a 24-well plate. Albendazole dilutions were prepared by dissolving 400 mg Zentel™ tablets (GlaxoSmithKline, London, UK) in 1% dimethyl sulfoxide (DMSO) (Sigma Aldrich, MO, USA). The final concentrations used for the egg hatch test were 0.05 ng/µl, 0.1 ng/µl, 0.5 ng/µl, 1.0 ng/µl, and 2.0 ng/µl. Each concentration was tested in triplicates, with controls containing 1% DMSO included. The 24-well plate was incubated at 27 °C for 48 h to allow egg hatching. After incubation, 10 µl of Lugol’s iodine was added to each well, and the total number of eggs and larvae was counted under an inverted microscope.

#### *Phenotypic resistance detection of levamisole via larva development test*

Following a modified protocol from Chagas et al. (2016) and Araújo-Filho et al. (2021) [38, 39], approximately 80 to 100 eggs suspended in 289 µl of water were added to each well of a 24-well plate. The plates were incubated at 27 °C for 24 h to allow egg hatching. After 24 h, 5 µl of yeast suspension dissolved in 0.85% NaCl and 6 µl of levamisole dilutions were added to each well. Levamisole hydrochloride power (Sigma Aldrich, MO, USA) was dissolved in 1% DMSO (Sigma Aldrich, MO, USA) to prepare final concentrations to 0.5 ng/µl, 1.0 ng/µl, 2.0 ng/µl, 10.0 ng/µl, and 20 ng/µl. Each drug concentration was tested in triplicates, with control wells containing 1% DMSO. The plates were then incubated for an additional 6 days. After incubation, 10 µl Lugol’s iodine was added to each well to terminate the assay. The total number of eggs, first-stage larvae (L1), and third-stage larvae (L3) were counted under an inverted microscope.

#### *Genotypic resistance detection of albendazole and levamisole via allele-specific SYBR green qPCR*

L3 larvae were collected using the Baermann technique [40], and genomic DNA was extracted from larval pools (per farm) following the previously described protocol. To detect albendazole resistance associated with the TAC mutation at F200Y of the β-tubulin gene in *Haemonchus* sp. and *Trichostrongylus* sp., a qPCR assay was performed using primers from Silvestre and Humbert (2000) [14]. For *Haemonchus* sp., the primers targeting the resistant-specific allele were Ph2 (5’-GATCAGCATTCAGCTGTCCA-3’) and Ph3 (5’-TGGTAGAGAACACCGATGAAACATA-3’), while the primers for the susceptible-specific allele were Ph1 (5’-GGAACGATGGACTCCTTTCG-3’) and Ph4 (5’-ATACAGAGCTTCGTTGTCAATACAGA-3’). For *Trichostrongylus* sp., the primers targeting the resistant-specific allele were Pc2 (5’-GGGAATCGGAGGCAAGTCGT-3’) and Pc3 (5’-CTGGTAGAGAATACCGATGAAACATA-3’), while the primers for the susceptible-specific allele were Pc1 (5’-GGAACAATGGATTCCGTTCG-3’) and Pc4 (5’-ATACAGAGCTTCGTTATCGATGCAGA-3’). qPCR assays were conducted separately for each primer pair to distinguish between resistant and susceptible alleles.

To detect levamisole resistance in *Haemonchus* sp. associated with the 63 bp deletion in the *Hco-acr-8b* gene, a qPCR assay was performed following the primers and protocol described by Santos et al. (2019) [41]. The forward primer LevF2 (5’-TAACCTTACCTATACACCCGTC-T-3’) was used for both resistant and susceptible-specific alleles. The reverse primer LevResR2 (5’-ATCTTTGACAGTAATCAGCGTTG-3’) was used to detect the resistant allele, while LevSusR2 (5’-CGGCGATATAACAGCAGTTAAC-3’) was used to detect the susceptible allele. qPCR assays were conducted separately for each primer pair to differentiate between resistant and susceptible alleles.

All qPCR reactions were performed using a CFX96 Touch™ Real-Time PCR system (Bio-Rad, California, USA). Each reaction was carried out in a final volume of 20 µl, comprising 10 µl of the Luna^®^ Universal qPCR master mix (New England Biolabs, MA, USA), 10 µM of each primer set, and 5 µl of gDNA template. The thermocycling conditions included an initial denaturation at 95 °C for 60 s, followed by 45 cycles of 95 °C for 15 s and 60 °C for 30 s. A final melt curve analysis was performed from 60 °C to 95 °C, with a 0.5 °C increment per cycle. Additionally, amplicons were visualized on a 1.5% agarose gel to assess primer formation and non-specific amplification.

#### *Data analysis*

For the phenotypic assays, the percentage of hatched eggs and ED_50_ values were calculated for EHT, while the percentage of larvae developing to the L3 stage and LD_50_ values were determined for the LDT. Log-probit analysis was performed using MedCalc version 22 [42]. Albendazole resistance was defined by an ED_50_ value exceeding 0.1 ng/µl, while levamisole resistance was indicated by an LD_50_ value above 2 ng/µl.

For genotype detection, the percentage of farms in each district with larval pools exhibiting homozygous resistant (RR), homozygous susceptible (SS), and heterozygous (RS) genotypes were calculated. Farms classified as RR showed amplification only with the resistant primer set, while SS farms showed amplification only with the susceptible primer set. RS farms exhibited amplification with both primer sets. Additionally, the resistant and susceptible allele frequencies were calculated for each district by determining the number of occurrences of each allele in the population and dividing by the total number of alleles.

## Results

### Identification, prevalence, and intensity of gastrointestinal nematodes

**Fig. 2:**
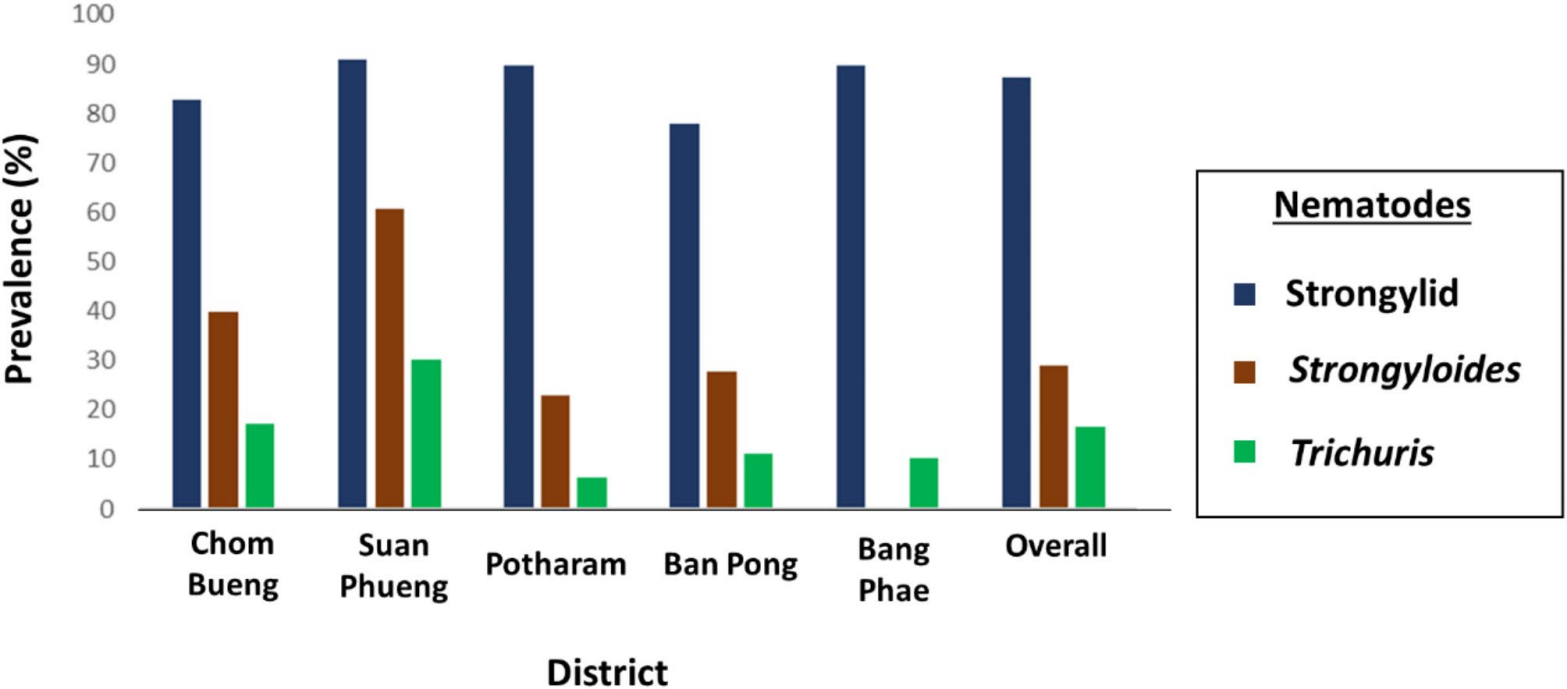
Prevalence of gastrointestinal nematode infection in goats

The fecal analysis of goat samples identified eggs of strongylids, *Strongyloides* sp., and *Trichuris* sp. Strongylids exhibited the highest overall prevalence at 87%, followed by *Strongyloides* sp. and *Trichuris* sp. Strongylids were detected across all five districts, with the highest prevalence (90%) recorded in Suan Phueng. *Trichuris* sp. was also present in all districts, while *Strongyloides* sp. was found in Chom Bueng, Suan Phueng, Potharam, and Ban Pong. Figure 2 illustrates the prevalence of gastrointestinal nematode infections in goats from these districts. In addition to the high prevalence of strongylid infections, the overall mean EPG count was 670 (Table 2). The highest EPG was recorded in farm in Potharam, with a mean of 6,125 EPG. Potharam had the highest mean EPG, followed by Bang Phae.

**Table 2:** Intensity of strongylid infection in goats

District	Farm	No. of samples	Egg per gram (mean ± SD)	Min - max
Chom Bueng	1	4	75 ± 86.6	0–150
	2	3	183 ± 202.1	0–400
	3	7	200 ± 370.8	0–950
	4	6	75 ± 75.8	0–200
	5	9	44 ± 76.8	0–200
	6	6	800 ± 273.9	700–1350
	**Total**	**35**	**225 ± 338.3**	**0–1350**
Suan Phueng	1	5	340 ± 248.5	0–550
	2	6	650 ± 655.0	100–1750
	3	3	433 ± 76.4	350–500
	4	8	568 ± 701.0	0–2050
	5	8	256 ± 417.8	0–1150
	6	3	316 ± 284.3	0–550
	**Total**	**33**	**437 ± 499.7**	**0–2050**
Potharam	1	10	6125 ± 5781.3	50–19,650
	2	7	121 ± 99.4	50–300
	3	3	0	0
	4	10	75 ± 100.7	0–250
	5	10	625 ± 747.3	0–1900
	6	8	481 ± 611.8	50–1900
	**Total**	**48**	**1519 ± 3509.3**	**0–19,650**
Ban Pong	1	3	16 ± 28.9	0–50
	2	1	100	100
	3	2	250 ± 0	250
	4	1	700	700
	5	5	10 ± 22.4	0–50
	6	6	275 ± 258.4	0–600
	**Total**	**18**	**169 ± 230.2**	**0–700**
Bang Phae	1	3	50 ± 86.6	0–150
	2	5	200 ± 187.1	50–450
	3	8	1406 ± 751.4	350–2250
	4	11	436 ± 262.8	100–800
	5	7	7 ± 18.9	0–50
	6	5	90 ± 74.2	0–200
	**Total**	**39**	**453 ± 627.3**	**0–2250**
Overall		173	670 ± 1953.3	0–19,650

### Molecular identification of strongylids

**Fig. 3:**
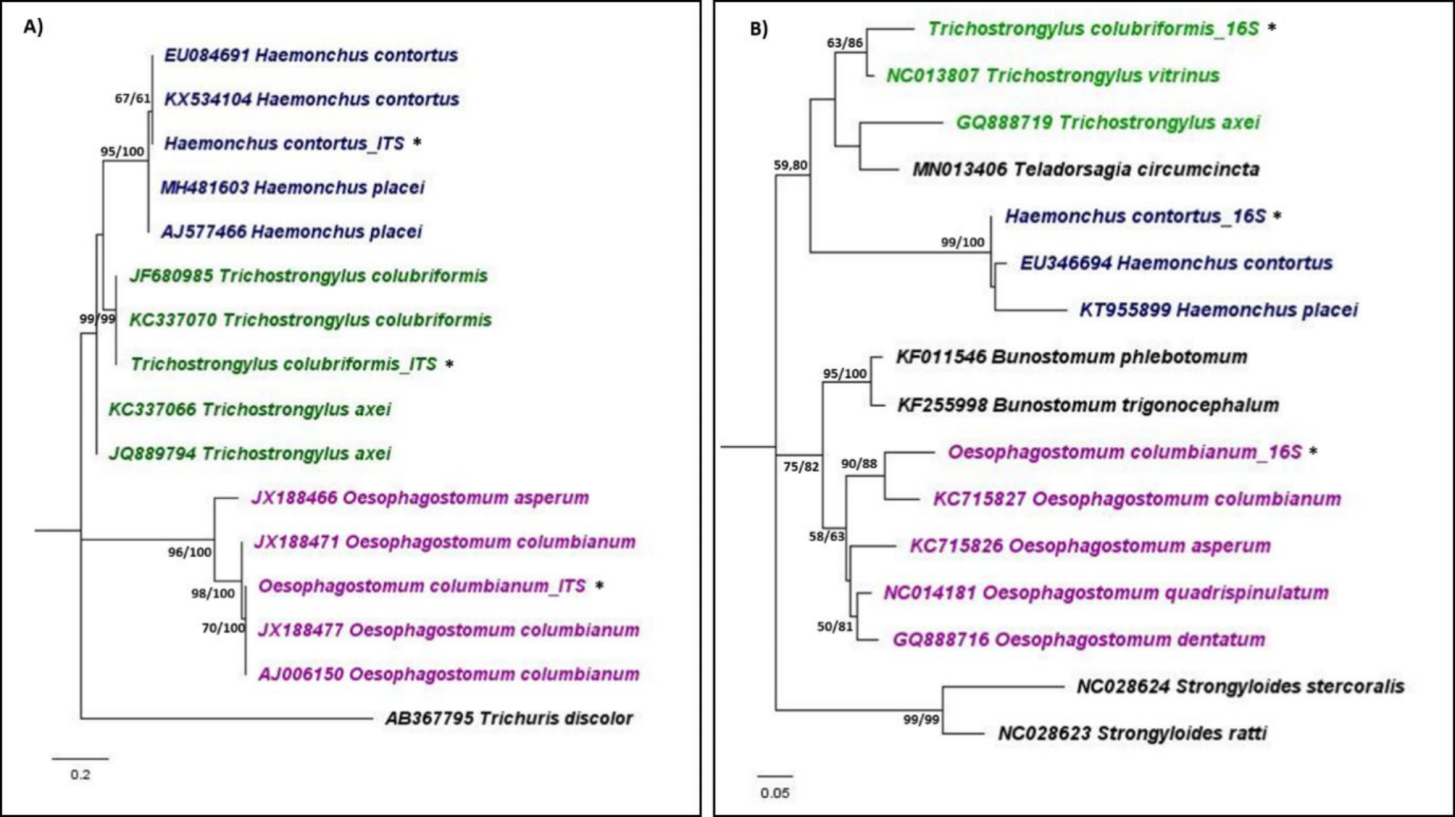
Maximum likelihood phylogeny using the **A**) nuclear ITS2 region (K2) and **B**) mitochondrial 16 S rRNA gene (TN + G). Numbers at nodes indicate bootstrap values (ML/NJ). Representative sequences generated from this study are indicated with an ‘*’

Strongylids were identified using the nuclear ITS2 region and the mitochondrial 16 S rRNA gene, confirming the presence of *Haemonchus* sp., *Trichostrongylus* sp., and *Oesophagostomum* sp. (Table 3). *Haemonchus* sp. was the most prevalent, detected in all farms (100%) positive for strongylid eggs. *Trichostrongylus* sp. was present in farms across all districts, comprising 96% of the strongylid-positive samples, while *Oesophagostomum* sp. was found in farms from Chom Bueng, Potharam, and Ban Pong. DNA sequencing of representative samples further identified the strongylids as *H. contortus*, *Trichostrongylus colubriformis*, and *Oesophagostomum columbianum*. The phylogenetic analysis based on the ITS2 region revealed that the representative sequences were 100% identical to *H. contortus*, *T. colubriformis*, and *O. columbianum*. *Haemonchus contortus* clustered in a monophyletic clade with *H. placei*, while *O. columbianum* formed a distinct monophyletic clade with other *Oesophagostomum* species. Phylogenetic analysis of the 16 S rRNA gene further supported these molecular identifications, with *H. contortus*, *O. columbianum*, and *T. colubriformis* sequences closely related to other species within their respective genera. Figure 3 illustrates the molecular phylogeny obtained.

**Table 3:** Percentage of strongylid genera detected in each district based on the nuclear ITS2 region and the mitochondrial 16 S rRNA gene

District	Genera		
	*Haemonchus*	*Trichostrongylus*	*Oesophagostomum*
**Chom Bueng**	100.00	83.33	33.33
**Suan Phueng**	100.00	100.00	0
**Potharam**	100.00	100.00	66.66
**Ban Pong**	100.00	100.00	40.00
**Bang Phae**	100.00	100.00	0
**Overall**	100.00	96.67	28.00

### Albendazole resistance status of strongylids infecting goats

EHT results from 20 farms revealed that 90% harbored resistant strongylid populations (Table 4). Moreover, resistant strongylid populations were detected in farms across all five districts. The ED_50_ values for resistant strongylid populations ranged from 0.23 ng/µl to 2.85 × 10^21^ mg/µl, with several values significantly exceeding 0.1 ng/µl, indicating high resistance levels. Among the 18 farms with resistant strongylid populations, 16 exhibited more than 90% eggs hatching at the discriminating dose of 0.1 ng/µl, further confirming the substantial degree of albendazole resistance in the strongylids infecting goats.

**Table 4:** Albendazole and levamisole resistance status determined by egg hatch test and larva development test, respectively

District	Farm	Albendazole (EHT)			Levamisole (LDT)		
		Resistance status	ED_50_	% hatch at DC^a^	Resistance status	LD_50_	% of L1 develop to L3 at DC^a^
Chom Bueng	2	Resistant	0.23 ng/µl	40	-	-	-
	6	Resistant	3.06 × 10^20^ mg/µl	98	Susceptible*	0.09 ng/µl	0
Suan Phueng	1	Resistant	0.26 ng/µl	47	-	-	-
	2	Resistant	1144 ng/µl	100	-	-	-
	3	Susceptible	0.001 ng/µl	41	-	-	-
	4	Susceptible*	0.029 ng/µl	21	Resistant	2268.2 ng/µl	56
Potharam	1	Resistant	117 mg/µl	91	Resistant	33.3 ng/µl	59
	2	Resistant	30 mg/µl	93	Resistant	127.35 ng/µl	63
	3	Resistant	836 mg/µl	98	-	-	-
	4	Resistant	91 ng/µl	100	Resistant	3.37 ng/µl	68
	5	Resistant	1040 mg/µl	98	-	-	-
	6	Resistant	768 mg/µl	99	-	-	-
Ban Pong	1	Resistant	22 × 10^9^ mg/µl	97	-	-	-
	2	Resistant	29 × 10^6^ mg/µl	99	-	-	-
	3	Resistant	12 × 10^4^ mg/µl	96	-	-	-
	4	Resistant	21 × 10^4^ mg/µl	91	-	-	-
	6	Resistant	38 × 10^7^ mg/µl	90	Resistant	3.54 ng/µl	73
Bang Phae	2	Resistant	7.28 × 10^6^ mg/µl	97	-	-	-
	3	Resistant	2.85 × 10^21^ mg/µl	97	Susceptible*	0.78 ng/µl	8
	6	Resistant	1.28 × 10^4^ mg/µl	100	-	-	-

A dash (-) indicates no data was obtained due to insufficient eggs collected. An asterisk (*) indicates that the genotype result obtained contrasted with the phenotype resistance status

^a^DC (discriminating dose) indicates the dose of the drug that is supposed to result in the mortality of the target organism. The DC indicating albendazole resistance is > 0.1 ng/µl, while for levamisole is > 2 ng/µl

**Fig. 4:**
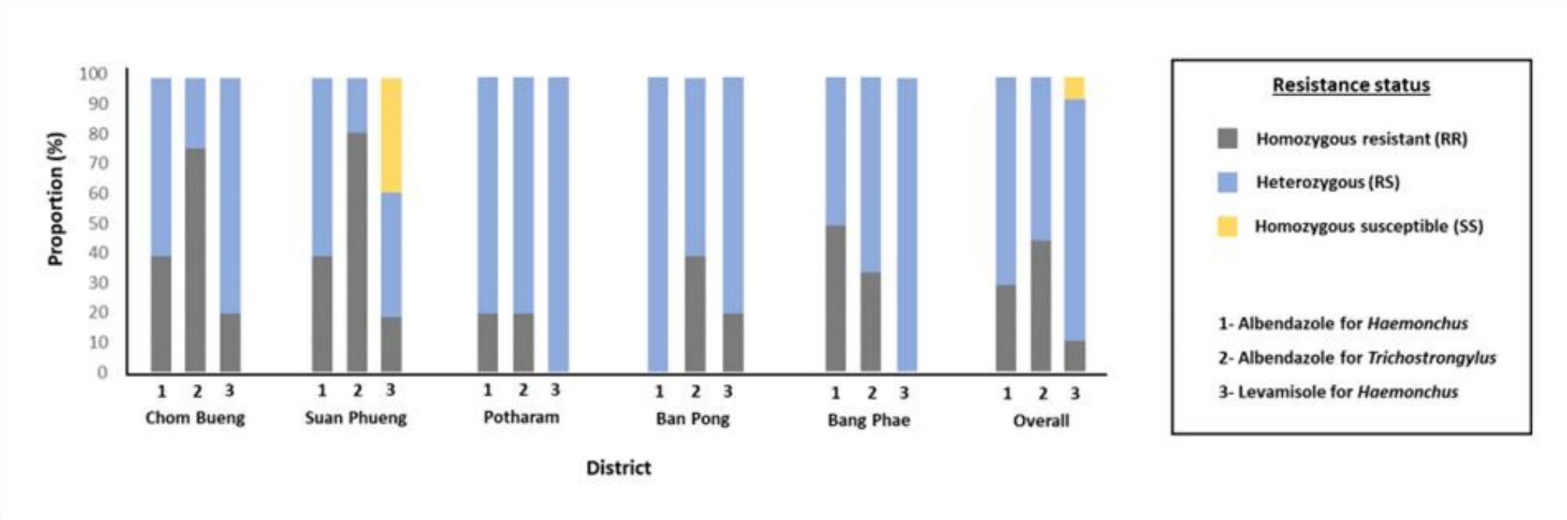
Genotypic resistance status for albendazole (*Haemonchus* sp. and *Trichostrongylus* sp.) and levamisole (*Haemonchus* sp.) for each district. The stacked bar chart represents the proportion of farms per district that were either RR, RS, or SS for albendazole (*Haemonchus* sp. and *Trichostrongylus* sp.) and levamisole (*Haemonchus* sp.)

Consistent with the phenotypic EHT results, the genotypic analysis revealed that 100% of the larval pools were resistant to albendazole for *Haemonchus* sp. and *Trichostrongylus* sp. Figure 4 illustrates the proportion of farms per district with homozygous resistant (RR), heterozygous (RS), and homozygous susceptible (SS) genotypes. For *Haemonchus* sp., 31% of the farms exhibited the RR genotype, while 69% were RS, In contrast, *Trichostrongylus* sp. had a higher proportion of RR genotypes, with 48% of the farms classified as RR and 52% as RS. The highest proportion of *Trichostrongylus* sp. RR genotypes were observed in Suan Phueng district (80% of farms), whereas *Haemonchus* sp. showed the highest RR proportion in Bang Phae district (50% of farms). The overall resistant allele frequency in the *Haemonchus* sp. population was 0.65, while the susceptible allele frequency was 0.35. Similarly, the *Trichostrongylus* sp. population exhibited a resistant allele frequency of 0.74 and a susceptible allele frequency of 0.26. Table 5 provides a detailed breakdown of resistant and susceptible allele frequencies in the population.

**Table 5:** Resistant and susceptible allele frequencies among the *Haemonchus* sp. and *Trichostrongylus* sp. populations in Ratchaburi Province

District/allele	Albendazole (*Haemonchus*)		Albendazole (*Trichostrongylus*)		Levamisole (*Haemonchus*)	
	Resistant	Susceptible	Resistant	Susceptible	Resistant	Susceptible
Chom Bueng	0.70	0.30	0.88	0.12	0.60	0.40
Suan Phueng	0.70	0.30	0.90	0.10	0.40	0.60
Potharam	0.60	0.40	0.60	0.40	0.50	0.50
Ban Pong	0.50	0.50	0.70	0.30	0.60	0.40
Bang Phae	0.75	0.25	0.67	0.33	0.50	0.50
Overall	0.65	0.35	0.74	0.26	0.52	0.48

### Levamisole resistance status of strongylids infecting goats

Among the seven farms tested for levamisole resistance using the LDT, five (71%) harbored strongylid populations resistant to levamisole (Table 4). Levamisole-resistant strongylid populations were detected in Suan Phueng, Potharam, and Ban Pong districts. The LD_50_ values for resistant strongylid populations ranged from 3.37 ng/µl to 2268.20 ng/µl.

Genotype screening for levamisole resistance in *Haemonchus* revealed that 92% of the 26 farms carried resistant alleles (RR or RS), while only 8% had the homozygous susceptible genotype (Fig. 4). Levamisole-susceptible farms (40%) were identified exclusively in Suan Phueng, whereas all farms in the remaining four districts exhibited levamisole resistance. Heterozygous (RS) farms were predominant in Potharam and Bang Phae, while homozygous resistant (RR) farms were detected in Chom Bueng, Suan Phueng, and Ban Pong. The overall frequency of the levamisole-resistant allele was 0.52, whereas the susceptible allele frequency was 0.48 (Table 5).

Additionally, genotypic screening of drug resistance demonstrated greater sensitivity than phenotypic methods. For albendazole and levamisole, molecular analysis identified a higher percentage of farms with resistant strongylid populations than phenotypic tests. Farms initially classified as susceptible based on phenotypic assays were later confirmed as resistant (RR or RS genotypes) through genotypic screening. For example, farm number 4 in Suan Phueng district tested resistant to albendazole upon genotypic analysis, while farm number 6 in Chom Bueng and farm number 3 in Bang Phae were found to be resistant to levamisole despite being classified as susceptible in phenotypic tests.

## Discussion

Morphological and molecular analyses revealed a high prevalence of GINs, particularly strongylids, infecting goats in Ratchaburi Province. Dual resistance of strongylids to benzimidazole (albendazole) and imidazothiazole (levamisole) was confirmed through both phenotypic and genotypic methods. Notably, this study provides the first molecular evidence of levamisole resistance in strongylids infecting goats in Thailand.

A high prevalence (87%) of strongylid infection was observed in goats from Ratchaburi Province, highlighting the significant threat these nematodes pose to goat health and farm productivity. In contrast, a previous study conducted in Ratchaburi Province between 2018 and 2019 reported a lower prevalence, with *Haemonchus* sp. and *Trichostrongylus* sp. detected at 18% and 11%, respectively [43]. The observed increase in prevalence may be attributed to factors such as the importation of goats from other provinces or the declining efficacy of anthelmintic drugs in recent years. In addition to strongylids, other GINs, including *Strongyloides* and *Trichuris*, were identified, further confirming the diversity of nematode infections in goats. Similarly, Junsri et al. (2021) also reported the presence of *Strongyloides* and *Trichuris* in goat farms in Ratchaburi Province [43].

A high prevalence of strongylid infection in goats has been reported in southern Thailand, particularly in Satun, Songkhla, Pattalung, Yala, Pattani, and Narathiwat Provinces, with prevalence ranging from 40 to 84.6% [19, 44]. Beyond the southern region, strongylid infections are also prevalent in the northeastern and central Provinces, including Nakhon Pathom, Samut Sakhon, Samut Songkhram, Petchaburi, Kanchanaburi, Khon Kaen, and Phitsanulok provinces [20, 21, 45, 46]. Congruent with our findings, *Haemonchus* sp. and *Trichostrongylus* sp. are the predominant strongylid genera infecting goats in Thailand, both of which are highly pathogenic [20, 44]. The high prevalence of these nematodes underscored the crucial role of veterinarians in assisting farmers in maintaining optimal animal health and farm productivity. Beyond their impact on goats health, *T. colubriformis* infections have also been reported in humans in Khon Kaen, Roi Et, Loei, and Sakon Nakhon Provinces [10, 47]. These zoonotic risks should not be overlooked, especially in goat farms where human and animal populations frequently coexist nearby (residence and farming areas are often nearby).

The emergence of anthelmintic resistance poses a significant threat to the health and economic sustainability of the goat farming industry, hindering effective control and prevention of helminthic infections [12, 48]. In Thailand, albendazole resistance in adult *H. contortus* infecting goats has been reported across five regions, with the highest resistance levels observed in the southern provinces [22]. Moreover, multiple drug resistance has been documented in various parts of the country [23, 25, 49]. Aside from albendazole resistance, ivermectin resistance has been reported in goat herds in Sing Buri, Khon Kaen, and Chaiyaphum Provinces through phenotypic tests [23, 49]. In Ratchaburi Province, albendazole and ivermectin resistance was detected in 81% (9/11) of goat farms using the FECRT [25]. As no molecular assays have been developed to detect ivermectin resistance, phenotypic tests remain the primary diagnostic method. Our molecular analysis based on larval pools revealed widespread dual drug resistance (albendazole and levamisole) across most farms. Albendazole resistance was detected in all farms. In contrast, phenotypic data reported by Paduang and Thongtha (2020) indicated no levamisole resistance among the sampled farms in Ratchaburi Province [25].

Widespread reports of anthelmintic resistance across all three drug classes, along with supporting evidence from various regions in Thailand, raise significant concerns. Without stringent regulation of anthelmintic drug use and goat movement between provinces, the local goat industry could face severe consequences. Moreover, Thailand plays a crucial role in the global goat trade, importing and exporting to and from Southeast Asia, Africa, the United States, and Australia. Within the country, benzimidazoles and macrocyclic lactones are the most commonly administered anthelmintic drugs for GIN infections in small ruminants, used by both farmers and veterinarians [50]. According to drug usage data from goat farmers in this study, albendazole and ivermectin were the primary anthelmintics used, with levamisole being administered only in Chom Bueng district. However, despite its limited use, levamisole resistance was still detected. A possible contributing factor to the spread of resistance is the movement of goats between regions. The goats examined in this study were not solely from Ratchaburi Province but were also sourced from other provinces in the Northeast, Western, and Central parts of Thailand. The importation and interprovincial movement of goats may facilitate the dissemination of drug-resistant alleles, exacerbating anthelmintic resistance. Consequently, the efficacy of anthelmintic treatments for GIN infections in goats will continue to decline, leading to an increased frequency of resistant alleles in strongylid population.

The small sample size (*n* = 7 farms) limited the detection of levamisole resistance via the LDT due to insufficient strongylid eggs. Priority was given to albendazole resistance detection using the EHT. Consequently, the reduced sample size may have inflated the final percentage of levamisole-resistant farms. Additionally, in vivo methods such as FECRT were not performed to assess drug efficacy. Resistance detection was conducted for genotype analysis using pooled larval samples per farm rather than individual worms due to the limited DNA yield from single larvae. As a result, the proportion for albendazole and levamisole resistance genotypic statuses and allele frequencies was based on the pooled larval samples rather than individual worms. Moreover, genotypic detection of levamisole resistance was only performed for *Haemonchus* species. Lastly, a lack of centrifugation step during fecal homogenization could have led to the loss of strongylid eggs and underestimate true parasite load.

## Conclusion

This study presents the first genotypic evidence of levamisole resistance in goats and is the first in Thailand to integrate both phenotypic and genotypic methods for anthelmintic resistance detection. Albendazole- and levamisole-resistant strongylid populations were identified in majority of the farms through phenotypic testing, with genotypic analysis further confirming resistance in *H. contortus* and *T. colubriformis* populations. The high prevalence of strongylid nematodes infecting goats in Ratchaburi Province, coupled with dual resistance to albendazole and levamisole, underscores the urgent need for alternative and sustainable parasite control strategies. Future studies should focus on monitoring resistance trends through longitudinal research, elucidating resistance mechanisms, and evaluating alternative anthelmintic compounds to ensure effective helminth control and mitigate anthelmintic resistance in goats.

## Electronic supplementary material

Below is the link to the electronic supplementary material.

Supplementary Material 1

## Data Availability

The dataset supporting the conclusions of this article is included within the article and its additional file.

## Abbreviations

DC: Discriminating dose
EHT: Egg hatch test
EPG: Eggs per gram
FECRT: Fecal egg count reduction test
GIN: Gastrointestinal nematode
ITS2: Internal transcribed spacer 2
LDT: Larval development test
ML: Maximum-likelihood
NJ: Neighbor-joining
qPCR: Quantitative PCR
rRNA: Ribosomal RNA
SNP: Single nucleotide polymorphism
