# Supplementary material for: Emergence of dual drug-resistant strongylids in goats: first phenotypic and genotypic evidence from Ratchaburi Province, central Thailand
Source: BMC Vet Res. 2025 Apr 4;21:245. doi: 10.1186/s12917-025-04700-4 (PMC11969839; doi:10.1186/s12917-025-04700-4)
Supplement: Supplementary file 1 — Supplementary Material 1 [file 12917_2025_4700_MOESM1_ESM.docx]

**Additional file 1. List of NCBI sequences used**

| **Genetic marker** | **Species** | **NCBI accession number** |
| --- | --- | --- |
| ITS2 | *Haemonchus contortus* | EU084691 |
|  |  | KX534104 |
|  |  | This study |
|  | *Haemonchus placei* | MH481603 |
|  |  | AJ577466 |
|  | *Trichostrongylus colubriformis* | JF680985 |
|  |  | KC337070 |
|  |  | This study |
|  | *Trichostrongylus axei* | KC337066 |
|  |  | JQ889794 |
|  | *Oesophagostomum asperum* | JX188466 |
|  | *Oesophagostomum columbianum* | JX188471 |
|  |  | JX188477 |
|  |  | AJ006150 |
|  |  | This study |
|  | *Trichuris discolor* (outgroup) | AB367795 |
| 16S rRNA | *Haemonchus contortus* | EU346694 |
|  |  | This study |
|  | *Haemonchus placei* | KT955899 |
|  | *Trichostrongylus colubriformis* | This study |
|  | *Trichostrongylus vitrinus* | NC013807 |
|  | *Trichostrongylus axei* | GQ888719 |
|  | *Teladorsagia circumcincta* | MN013406 |
|  | *Bunostomum phlebotomum* | KF011546 |
|  | *Bunostomum trigonocephalum* | KF255998 |
|  | *Oesophagostomum columbianum* | This study |
|  |  | KC715827 |
|  | *Oesophagostomum asperum* | KC715826 |
|  | *Oesophagostomum quadrispinulatum* | NC014181 |
|  | *Oesophagostomum dentatum* | GQ888716 |
|  | *Strongyloides stercoralis* (outgroup) | NC028624 |
|  | *Strongyloides ratti* (outgroup) | NC028623 |
